# R2R3-MYB transcription factor GEMMA CUP-ASSOCIATED MYB1 mediates the cytokinin signal to achieve proper organ development in *Marchantia polymorpha*

**DOI:** 10.1038/s41598-022-25684-3

**Published:** 2022-12-07

**Authors:** Shiori S. Aki, Tomoyo Morimoto, Taiki Ohnishi, Ayumi Oda, Hirotaka Kato, Kimitsune Ishizaki, Ryuichi Nishihama, Takayuki Kohchi, Masaaki Umeda

**Affiliations:** 1 Graduate School of Science and Technology, Nara Institute of Science and Technology, Takayama 8916-5, Ikoma, Nara 630-0192 Japan; 2 Graduate School of Science, Kobe University, Kobe, Hyogo 657-8501 Japan; 3 Department of Applied Biological Science, Faculty of Science and Technology, Tokyo University of Science, Yamazaki 2641, Noda, Chiba 278‐8510 Japan; 4 Graduate School of Biostudies, Kyoto University, Kyoto, 606-8502 Japan; 5 Present Address: Graduate School of Science and Engineering, Ehime University, 2-5, Bunkyo-Cho, Matsuyama, Ehime 790-8577 Japan

**Keywords:** Plant hormones, Plant molecular biology

## Abstract

Cytokinin, a plant hormone, plays essential roles in organ growth and development. The type-B response regulator-mediated cytokinin signaling is repressed by type-A response regulators and is conserved in the liverwort *Marchantia polymorpha.* Its signal coordinates the development of diverse organs on the thallus body, such as the gemma cup, rhizoid, and air pores. Here we report that the type-B response regulator MpRRB upregulates the expression of the R2R3-MYB transcription factor *GEMMA CUP-ASSOCIATED MYB1* (Mp*GCAM1*) in *M. polymorpha*. Whereas both Mp*gcam1* and Mp*rrb* knockout mutants exhibited defects in gemma cup formation, the Mp*gcam1* Mp*rra* double mutant, in which cytokinin signaling is activated due to the lack of type-A response regulator, also formed no gemma cups. This suggests that MpGCAM1 functions downstream of cytokinin signaling. Inducible overexpression of Mp*GCAM1* produced undifferentiated cell clumps on the thalli of both wild-type and Mp*rrb.* However, smaller thalli were formed in Mp*rrb* compared to the wild-type after the cessation of overexpression. These results suggest that cytokinin signaling promotes gemma cup formation and cellular reprogramming through MpGCAM1, while cytokinin signals also participate in activating cell division during thallus development.

## Introduction

The phytohormone cytokinin is involved in a broad range of physiological events, such as cell proliferation and differentiation, organ growth, and shoot initiation^1^. In plants, its signaling is mediated by a phosphorelay system, in which CHASE domain-containing histidine kinase receptors (CHKs) autophosphorylation is triggered by the cytokinin perception via CHK at the plasma and endoplasmic reticulum membrane. This is followed by a transfer of phosphate to cytosolic histidine-containing phosphotransfer proteins (HPTs). After moving into the nucleus, HPTs transfer the phosphate to type-B response regulators (RRs), which are then activated and function as transcription factors to regulate the expression of target genes^2–4^. One of the target genes encode type-A RRs that negatively control cytokinin signaling by competing with type-B RRs for phosphate transfer^5,6^. *Arabidopsis* RRs (ARRs) include 10 type-A and 11 type-B members, among which type-B RRs ARR1, ARR10, and ARR12 play a predominant role in transmitting cytokinin signals. Previous studies demonstrated that the *arr1 arr10 arr12* triple mutant largely lacked cytokinin-dependent gene expression and exhibited retarded shoot development, growth arrest of primary roots, and defects in seed enlargement^7–9^.

Liverwort *Marchantia polymorpha* is a model in bryophytes^10–12^. Its dominant plant body is a flattened thalloid and the haploid gametophyte, called thallus. On the dorsal side of thalli, the gemma cup, a cup-shaped receptacle, generates clonal progeny called gemmae, and air chambers constitute an intracellular space to strike a balance between gas exchange and water vapor loss^11^. On the ventral side, tubular cells called rhizoids function in anchorage to soil. These tissues are produced by highly regulated division of the apical cell and its descendants at the apical notch that resides at the thallus tip^11,13^.

*Marchantia polymorpha* possesses a minimal but complete set of genes for cytokinin signaling: one gene for each of type-A and type-B RRs, which were designated Mp*RRA* and Mp*RRB*, respectively^12,14,15^. We previously reported that the Mp*RRB* promoter activity was observed at the apical notch of young thalli^15^. Interestingly, transgenic plants defective in cytokinin signaling, such as Mp*rrb* knockout lines, formed no gemma cup and more rhizoids compared to the wild-type^15^. Furthermore, distribution of air pores and the shape of the thallus margin were impaired in the Mp*rrb* knockout line^16^. These observations indicate that tissue/organ formation in the thallus occurred due to the coordination of cytokinins regulating cell proliferation and/or differentiation of precursor cells derived from the apical cell. We previously reported that exogenous cytokinin application to the wild-type had limited effects on thallus development, and a high concentration of cytokinins (e.g., 50 µM) inhibited gemma cup and gemma formation, and thallus growth^15^. This is likely due to relatively high level of endogenous cytokinins in *M. polymorpha* because transgenic lines overexpressing Mp*CKX2*, which encodes a cytokinin oxidase that inactivates cytokinins^17^, had a clear phenotype, such as fewer or no gemmiparous cells, which are precursor cells that produce the gemma cup near the apical cell^15^.

The R2R3-MYB transcription factor GEMMA CUP-ASSOCIATED MYB1 (MpGCAM1) plays a crucial role in gemma cup formation^18^. Mp*GCAM1* is expressed at both the apical notch and in developing gemma cups, and its absence results in a lack of gemma cups. However, an overexpression of Mp*GCAM1* did not promote the formation of gemma cups but rather generated undifferentiated cell clumps, which had the potential to produce intact thalli^18^. This led to the hypothesis that MpGCAM1 maintains dorsal cells at the apical notch in an undifferentiated state, thereby ensuring gemma cup formation on the dorsal side^18,19^. MpGCAM1 is orthologous to REGULATOR OF AXILLARY MERISTEM (RAX) in *Arabidopsis* and Blind in tomato plants (*Solanum lycopersicum*), which function in axillary meristem formation^20–22^. Indeed, the defects observed in the *rax1 rax2 rax3* triple mutant were partly rescued by the expression of Mp*GCAM1*^18^.

Here we identified Mp*GCAM1* as a gene downstream of MpRRB-mediated cytokinin signaling. The expression of Mp*GCAM1* was responsive to exogenous cytokinin application and an increase in cytokinin signaling. Our genetic data further showed that MpGCAM1’s function in gemma cup formation is controlled downstream of cytokinin signaling, and that MpRRB is also involved in preserving proliferation activity at the apical notch during thallus development.

## Results

### Identification of downstream genes of MpRRB-mediated cytokinin signaling

**Figure 1:**
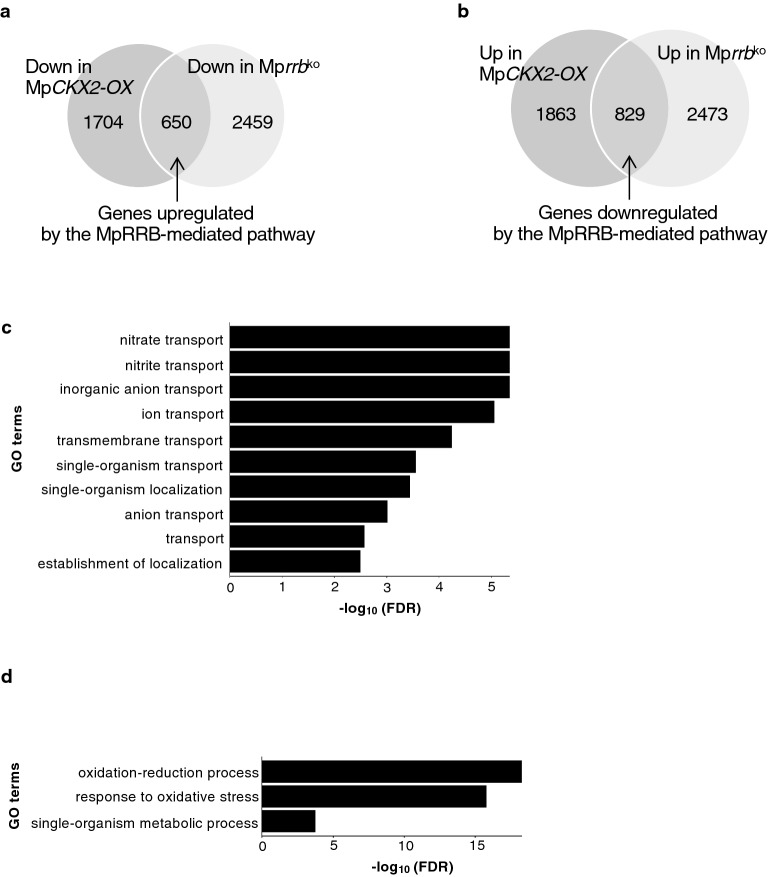
Identification of genes downstream of cytokinin signaling. (**a,b**) Venn diagrams overlap among genes with more than a (**a**) decreased or (**b**) increased transcript level of 1.5-fold in both the Mp*CKX2*-overexpressing line and the Mp*rrb* knockout line compared to the wild-type. (**c,d**) GO terms of gene sets (**c**) upregulated or (**d**) downregulated by MpRRB-mediated cytokinin signaling. Overlaps shown in (**a,b**) were subjected to GO enrichment analysis.

A ribonucleic acid-sequence (RNA-seq) analysis was performed to search for genes controlled downstream of MpRRB-mediated cytokinin signaling. Data from three biological replicates of the Mp*CKX2*-overexpressing line and the Mp*rrb* knockout line^15^ were used to identify genes with more than a 1.5-fold reduction in expression levels compared to the wild-type Tak-1 and Tak-2, respectively. Mp*RRA* mRNA levels in the two lines were 0.76- and 0.67-fold of those in the wild-type, respectively, indicating the reliability of the obtained data. It is notable that such a mild reduction in Mp*RRA* transcript levels was also observed in quantitative RT-PCR (qRT-PCR)^15^. The genes, whose expression was reduced in both Mp*CKX2*-overexpressing and Mp*rrb* knockout lines, were extracted as those upregulated by MpRRB-mediated cytokinin signaling (Fig. 1a). Conversely, genes that represented more than a 1.5-fold increase in expression levels in both lines were listed as those downregulated by the MpRRB-mediated pathway (Fig. 1b). As a result, 650 upregulated and 829 downregulated genes were identified (Tables S1, S2, respectively).

The GO enrichment analysis showed that genes associated with nitrate/nitrite transport were highly enriched among the upregulated genes (Fig. 1c). Eighteen upregulated genes were related to nitrate assimilation and transport as follows: one gene for nitrite reductase (orthologous to *Arabidopsis* NIR1), 12 genes for nitrate transporter (orthologous to NRT2), two genes for NRT2-associating protein (orthologous to NRT3.1), and three genes for ammonium transporter (orthologous to AMT1) (Table S1). Some of the genes were tested by qRT-PCR, which supported the transcriptional change observed in the RNA-seq data (Fig. S1). MpRRB-mediated upregulation of the *NIR1* ortholog may indicate a promotive effect of cytokinins on nitrate assimilation^23^. In *Arabidopsis*, the high-affinity nitrate transporter NRT2 is known to form a complex with NRT3.1 (also referred to as NAR2.1), enhancing nitrate absorption through the roots of plants^24^ and AMT1 functions in ammonium uptake^25^. Therefore, in *M. polymorpha*, cytokinins are necessary for the expression of *NRT2*, *NRT3.1*, and *AMT1* orthologs, and may promote the uptake of nitrate and ammonium from soil. The GO terms related to oxidative stress were significantly enriched in downregulated genes (Fig. 1d). Fifty-three downregulated genes encode peroxidase superfamily proteins (Table S2). Although cytokinins also downregulate several peroxidase genes in *Arabidopsis*^26^, the physiological role has not been identified thus far.

Mp*CKX1* and Mp*IPT2* were found to be listed in the upregulated and downregulated genes, respectively (Tables S1, S2). Mp*CKX1* is one of the two *CKX* genes in *M. polymorpha*, and Mp*IPT2* is orthologous to *Arabidopsis IPT9*, encoding a tRNA isopentenyltransferase (IPT) that catalyzes the addition of prenyl-moiety to a tRNA-bound adenine nucleotide and contributes to cytokinin biosynthesis^27^. *Arabidopsis CKX4* is also known to be induced by cytokinins^4,26,28^. Therefore, to adjust the hormonal level in plant bodies, it is likely conserved in land plants that cytokinin signaling promotes degradation and inhibits biosynthesis of cytokinins. It was observed that a few auxin-related genes displayed cytokinin responses. Namely, Mp*PIN-FORMED3* (Mp*PIN3*) encoding an auxin efflux carrier was induced by MpRRB-mediated cytokinin signaling, whereas Mp*GRETCHEN HAGEN 3* (*GH3*) *B* (Mp*GH3B)* was downregulated (Tables S1, S2). In *Arabidopsis*, *GH3* encodes an acyl acid amido synthetase, which catalyzes the conjugation reaction of indole-3-acetic acid (IAA) with amino acids, leading to inactivation of IAA^29^. Thus, to achieve continuous organ growth, *M. polymorpha* may regulate auxin homeostasis in response to cytokinins.

### Cytokinin signaling upregulates the expression of the R2R3-MYB transcription factor GCAM1

**Figure 2:**
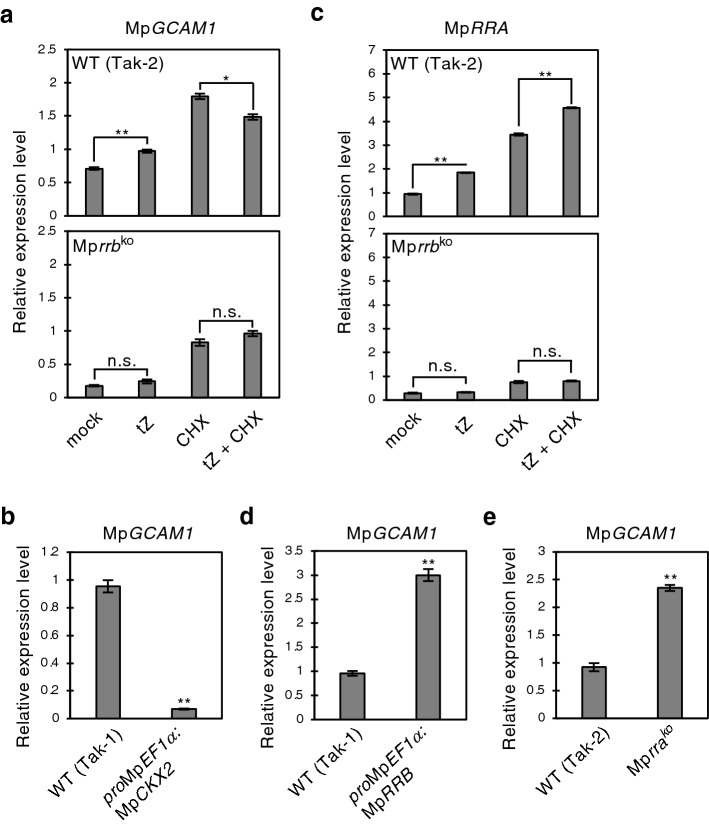
Mp*GCAM1* transcript levels in tZ-treated plants, Mp*CKX2*- or Mp*RRB*-overexpressing plants, and the Mp*rra* knockout mutant. (**a**) Transcript levels of Mp*GCAM1* in the wild-type (Tak-2) and the Mp*rrb* knockout line with or without 50 µM of tZ treatment and/or 10 µM of CHX for 3 h. (**b**) Transcript levels of Mp*GCAM1* in plants overexpressing Mp*CKX2*. (**c**) Transcript levels of Mp*RRA* in the wild-type (Tak-2) and the Mp*rrb* knockout line with or without 50 µM of tZ treatment and/or 10 µM of CHX for 2 h. Transcript levels of Mp*GCAM1* in (**d**) plants overexpressing Mp*RRB* and in the (**e**) Mp*rra* knockout line. mRNA levels were normalized to that of Mp*EF1α*. Data are presented as mean ± SD (*n* = 3). Significant differences from the plants with (**a,c**) mock or CHX treatment and from the (**b,d,e**) wild-type were determined by Student’s t-test as follows: *P < 0.05; **P < 0.01; *n.s.* not significant.

Among the upregulated genes downstream of MpRRB-mediated cytokinin signaling, we found Mp6g04830, a gene encoding the R2R3-MYB transcription factor MpGCAM1 (Table S1). Mp*gcam1* mutants had defects in gemma cup formation, and these defects were also observed in the Mp*rrb* knockout line^15,18^. This prompted further investigation of Mp*GCAM1* in terms of cytokinin signaling. The FPKM values of this gene in Mp*CKX2*-overexpressing and Mp*rrb* knockout lines were 0.64 and 0.76, respectively. This represents a 13.8- and 9.4-fold reduction compared to the expression levels in the wild-type Tak-1 and Tak-2, respectively. In support of the RNA-seq data, the qRT-PCR analysis showed that the Mp*GCAM1* transcript level in the Mp*rrb* line and the Mp*CKX2*-overexpressing line was 4.0- and 13.7-fold lower than that in the wild-type, respectively (Fig. 2a,b).

Next, we tested whether Mp*GCAM1* responds to exogenously applied cytokinins. When thallus tips of wild-type plants were treated with 50 µM of trans-zeatin (tZ), Mp*GCAM1* transcripts increased by more than 1.4-fold (Fig. 2a). However, such an increase was not observed in the Mp*rrb* line, and a similar trend was also seen for Mp*RRA* mRNA (Fig. 2a,c), indicating that Mp*GCAM1* as well as Mp*RRA* is induced by cytokinins through the MpRRB-mediated pathway. Furthermore, the Mp*GCAM1* mRNA level in the Mp*RRB*-overexpressing line and the Mp*rra* knockout line in which the cytokinin signaling is activated was 3.1- and 2.6-fold higher, respectively (Fig. 2d,e). These results support the idea that Mp*GCAM1* is controlled by cytokinin signaling.

To examine whether Mp*GCAM1* is a direct target of MpRRB, we treated wild-type plants with 10 µM of cycloheximide (CHX), a protein synthesis inhibitor, and conducted a qRT-PCR analysis. As shown in Fig. 2c, cytokinin-dependent induction of Mp*RRA* was observed even in the presence of CHX, suggesting that Mp*RRA* is directly upregulated through the MpRRB-mediated pathway. It is notable that the application of CHX alone increased Mp*GCAM1* and Mp*RRA* transcripts. This is likely due to an indirect effect of the inhibition of protein synthesis (Fig. 2a,c). Conversely, the level of Mp*GCAM1* mRNA slightly decreased as a result of the tZ treatment in the presence of CHX (Fig. 2a). This result suggests that in response to cytokinins, Mp*GCAM1* is not a direct target of MpRRB and requires de novo protein synthesis for its induction.

**Figure 3:**
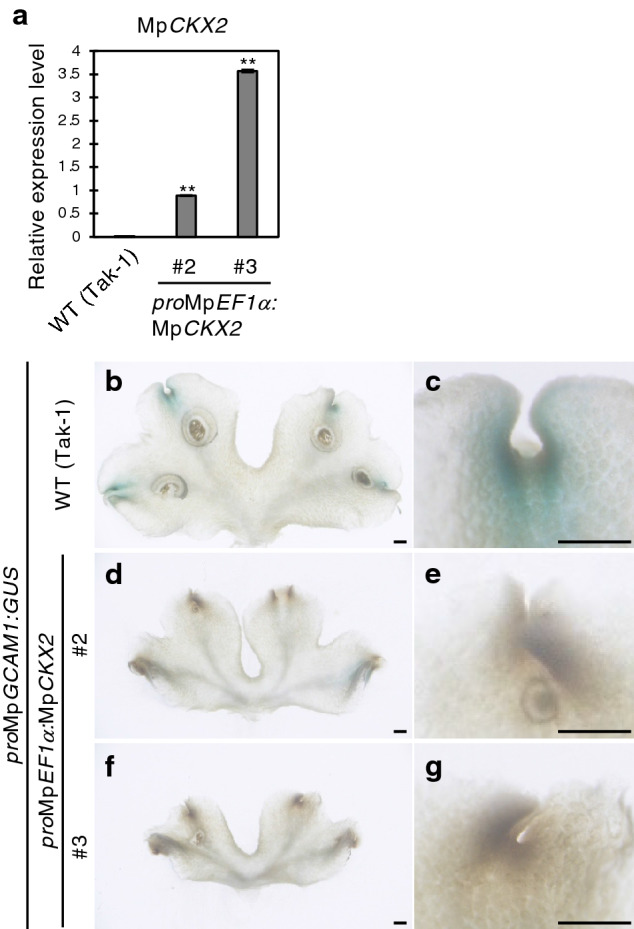
Expression pattern of Mp*GCAM1* in the Mp*CKX2*-overexpressing line. (**a**) Transcript levels of Mp*CKX2* in the wild-type (Tak-1) and Mp*CKX2*-overexpressing lines #2 and #3. mRNA levels were normalized to that of Mp*EF1α*. Data are presented as mean ± SD (*n* = 3). Significant differences from the wild-type were determined by the Student’s t-test as follows: **P < 0.01. (**b–g**) GUS staining of 20-day-old thalli carrying *pro*Mp*GCAM1:GUS*. Wild-type (Tak-1) and the Mp*CKX2*-overexpressing lines #2 and #3 were observed. Magnified images around the apical notch are shown (**c,e,g**). Bars represent 1 mm.

Yasui et al.^18^ previously constructed a β-glucuronidase (GUS)-reporter gene for Mp*GCAM1*, in which the 5215-bp upstream and 378-bp downstream from start codon of Mp*GCAM1* were fused in-frame to the *GUS* gene. In the transgenic line, the GUS signal was detected in the apical notch, the floor of gemma cups and developing gemma, a similar expression pattern to that of the Mp*RRBpro:GUS* line^15,18^. Mature thalli of Mp*GCAM1pro:GUS* were treated with 50 µM of tZ and used for histological GUS staining (Fig. S2). However, the signals neither increased nor spatially expanded upon tZ treatment. This is likely due to their limited sensitivity to exogenously applied cytokinins in *M. polymorpha,* as mentioned above^15^. To overcome this problem, the expression of Mp*GCAM1pro:GUS* in the Mp*CKX2*-overexpressing line containing a reduced amount of endogenous cytokinins was observed^15^. Five independent lines showed the same *GUS* expression pattern. Therefore, the representative lines #2 and #3 with different Mp*CKX2* expression levels were used for detailed analysis (Fig. 3a). GUS staining showed that the signals were missing at the apical notch in both lines (Fig. 3b–g), indicating that cytokinins upregulate the Mp*GCAM1* expression in the thallus tissues. The effect of cytokinins on Mp*GCAM1* expression in gemma cups could not be estimated since gemma cups were rarely formed in the Mp*CKX2*-overexpressing lines. However, the Mp*rra* knockout line produced equal quantities of gemma as observed in the wild-type (Fig. S3), while generating more gemma cups^15^, suggesting that the MpRRB-mediated cytokinin signaling has no or limited function in gemma production.

### Mp*GCAM1* is involved in cytokinin-dependent gemma cup formation

**Figure 4:**
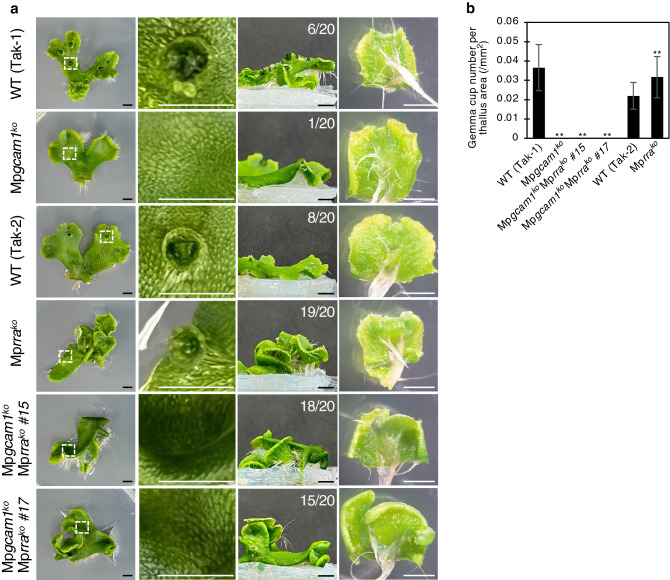
Morphological phenotype of the Mp*gcam1* Mp*rra* double mutant. (**a**) Thalli of the Mp*gcam1* and Mp*rra* single mutants, and the Mp*gcam1* Mp*rra* double mutant were observed from the top (left), from the side (second from the right), and from the bottom (right). Magnified images of gemma cups or predicted position for gemma cup formation are shown (second from the left). The numbers in the side views indicate the number of curled thalli per total thalli. Bars represent 5 mm. (**b**) Gemma cup number per thallus area. Thallus apices were cultured for 14 days. Data are represented as mean ± SD (*n* = 20). Significant differences from the wild-type (Tak-1 and Tak-2) were determined by the Student’s t-test as follows: **P < 0.01.

To examine genetic interaction between Mp*GCAM1* and cytokinin signaling, double knockout mutants were generated by crossing the male Mp*gcam1* line and the female Mp*rra* line. The double mutant lines #15 and #17 never formed gemma cups like the Mp*gcam1* line (Fig. 4a,b), implying that enhanced gemma cup formation caused by the defect in Mp*RRA* was suppressed in the Mp*gcam1* mutant. This result suggests that Mp*GCAM1* functions downstream of cytokinin signaling in terms of gemma cup formation. Curled thalli were produced in the Mp*rra* knockout line^15^, whereas Mp*gcam1* did not exhibit any curling phenotype (Fig. 4a). Interestingly, the Mp*gcam1* Mp*rra* double mutants also produced curled thalli (Fig. 4a), indicating that Mp*GCAM1* is dispensable for cytokinin-dependent differential growth of thalli. It is notable that rhizoid formation was comparable in the wild-type and single mutants as previously described^15,18^, and no difference was found between the wild-type and double mutants (Fig. 4a).

### Cytokinin signaling promotes thallus development from dedifferentiated cell clumps

**Figure 5:**
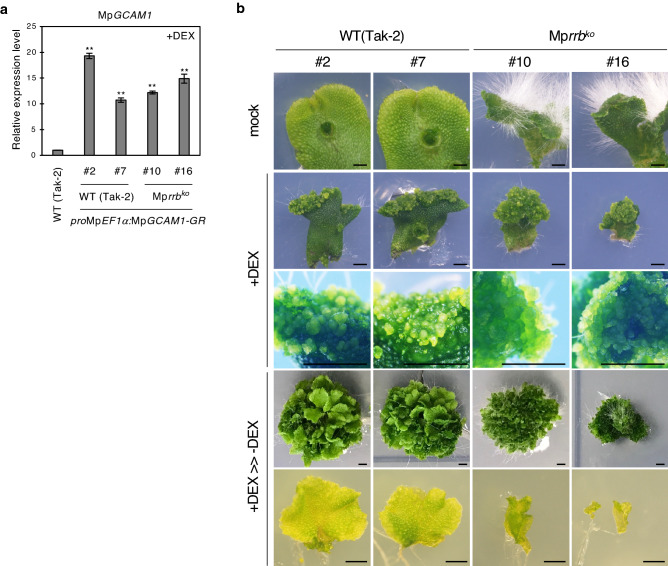
Morphological phenotype of the Mp*rrb* knockout lines overexpressing Mp*GCAM1*. (**a**) Transcript levels of Mp*GCAM1* in the wild-type (Tak-2) and Mp*rrb* that overexpress Mp*GCAM1*. Thalli were treated with 5 µM DEX for 14 days. mRNA levels were normalized to that of Mp*EF1α*. Data are presented as mean ± SD (*n* = 3). Significant differences from the wild-type (Tak-2) were determined by the Student’s t-test as follows: **P < 0.01. (**b**) Thalli of the wild-type and Mp*rrb*, which carry *pro*Mp*EF1α:*Mp*GCAM1–GR*, were treated with (+ DEX) or without (mock) 5 µM DEX for 14 days. The DEX-treated thalli were transferred onto a medium without DEX and were further cultured for 28 days (+ DEX > >  − DEX). The lower panels for DEX-treated and recovered samples are magnified images of cell clumps in the tip region and regenerated thalli, respectively. Bars represent 2 mm.

To investigate the role of cytokinin signaling in organ formation, we took advantage of the dedifferentiation phenotype caused by Mp*GCAM1* overexpression. Transgenic plants overexpressing Mp*GCAM1* with a dexamethasone (DEX)-inducible system in the wild-type and the Mp*rrb* knockout line were generated. MpGCAM1 fused to the hormone-binding domain of the glucocorticoid receptor remains inactive due to its cytoplasmic localization in the absence of DEX, while it moves to the nucleus and exerts the function in the presence of DEX^18^. Seven independent lines for the wild-type and nine independent lines for the Mp*rrb* line were isolated, and two lines were selected for each one with similar Mp*GCAM1* expression levels for further analysis (Fig. 5a). When plants were cultivated in the presence of 5 µM DEX, thallus growth was severely inhibited, and cell clumps and few rhizoids were generated mainly in the tip region of both wild-type and Mp*rrb* (Fig. 5b). This suggests that the overexpression of Mp*GCAM1* is sufficient to reprogram and produce dedifferentiated cells in the absence of cytokinin signaling. A number of small thalli were generated at random positions in the wild-type when the DEX-treated plants were transferred to a DEX-free medium. Similarly, small thalli were produced in the Mp*rrb* line, while the thallus size was much smaller than that of the wild-type (Fig. 5b). This result suggests that MpRRB-mediated cytokinin signaling is involved in activating cell proliferation after initiating thallus development in a DEX-free medium.

### Mp*GCAM1* regulates gemma cup formation without enhancing cytokinin biosynthesis

**Figure 6:**
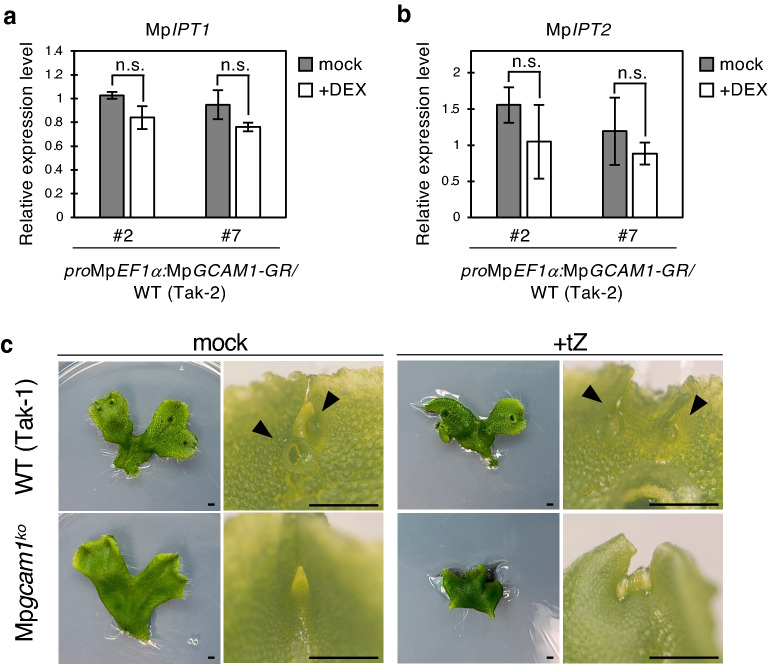
Mp*IPT* transcript levels in Mp*GCAM1*-overexpressing plants and Mp*gcam1* knockout lines grown in the presence of tZ. (**a,b**) Transcript levels of Mp*IPT1* (**a**) and Mp*IPT2* (**b**) in plants overexpressing Mp*GCAM1-GR*, which were treated with or without 10 µM DEX for 2 h. mRNA levels were normalized to that of Mp*EF1α*. Data are presented as mean ± SD (*n* = 3). Significant differences from the plants with mock treatment were determined by Student’s t-test. *n.s.* not significant. (**c**) Thallus apices of the wild-type (Tak-1) and Mp*gcam1* were treated with (+ tZ) or without (mock) 50 µM tZ for 14 days. The right panels are magnified images of apical notches. Arrowheads indicate gemma cups. Bars represent 2 mm.

Mp*GCAM1* is orthologous to *Arabidopsis RAX* genes, whose defects suppressed cytokinin signaling and impaired axillary meristem formation^18,21,22,30^. Exogenous cytokinin application or ectopic expression of the cytokinin biosynthesis gene *IPT8* could rescue the phenotype of the *rax1 rax2 rax3* triple mutant, which implied that *RAX*s regulated axillary meristem formation through enhancing cytokinin biosynthesis^31^. We first quantified the expression levels of Mp*IPT1* and Mp*IPT2,* the two cytokinin biosynthesis genes in *M. polymorpha,* in the two Mp*GCAM1-GR*-overexpressing lines to test the possibility of Mp*GCAM1* controlling cytokinin biosynthesis for generating gemma cups. Data showed that transcripts of neither Mp*IPT1* nor Mp*ITP2* increased after 2 h of 10 µM DEX treatment (Fig. 6a,b). We also treated thallus apices of the Mp*gcam1* mutant with 50 µM tZ for 14 days. However, de novo formation of gemma cups was never observed under our experimental conditions (Fig. 6c). These results suggest that Mp*GCAM1* controls gemma cup formation through pathway(s) that are independent of cytokinin biosynthesis, while it is under the control of cytokinin signaling.

## Discussion

In this study, we found that MpRRB upregulates genes encoding ammonium and nitrate transporters involved in nitrogen uptake (Fig. 1c, Table S1). This result is interesting because the expression of *NRT2*s and *NRT3.1* is not induced in *Arabidopsis*. Rather, it is reduced upon cytokinin application in roots^32^. Conversely, nitrogen promotes cytokinin biosynthesis by inducing the expression of the key biosynthesis gene *IPT3* in *Arabidopsis*^33,34^. This raises the possibility that nitrogen elevates the level of cytokinin in the plant body, while cytokinins restrict nitrogen uptake by repressing transporter genes. As a result, the cytokinin content maintains a proper level and prevents overgrowth under nitrogen-rich conditions. Although it remains unknown whether nitrogen induces cytokinin biosynthesis gene(s) in *M. polymorpha*, it is likely that cytokinin-triggered induction of transporter genes promotes nitrogen uptake and enhances de novo production of adenine derivatives including cytokinins. This may enable rapid and indeterminate growth of *M. polymorpha* and expand its territory on land. However, by applying negative feedback between nitrogen and cytokinins, flowering plants might have developed a mechanism to adjust their growth in response to environmental conditions.

The possible roles of hormones in gemma and gemma cup formation have been described for those other than cytokinins as well. For instance, the exogenous application of auxin inhibited gemma cup formation, whereas a slight reduction in the auxin level increased gemma cups^35,36^. Plants expressing the non-degradable form of the Aux/IAA transcriptional repressor, which inhibits auxin signaling, produced callus-like tissues that occasionally generated gemma cup clusters^37^. These observations suggest that, in contrast to cytokinins, auxin has a repressive function in gemma cup formation. Additionally, smoke-derived germination stimulants, karrikins, regulate gemma development through the KARRIKIN INSENSITIVE2 (KAI2) receptor-mediated pathway^38^. In this study, we revealed that MpRRB indirectly controls the Mp*GCAM1* expression, implying that other transcriptional regulator(s) mediate the signaling from MpRRB to Mp*GCAM1*. Considering that MpGCAM1 is involved not only in gemma and gemma cup formation but also in cellular reprogramming and maintaining the undifferentiated state, it is likely that the transcriptional regulator(s) downstream of MpRRB perceive distinct hormonal signals and control the development of multiple organs. It is intriguing to reveal how cytokinin and auxin cross-talk to fine-tune gemma cup and rhizoid formation, thereby coordinating reproduction and nutrient/water uptake to adapt to changing environments.

A previous report demonstrated that the overexpression of Mp*GCAM1* led to production of undifferentiated cell clumps^18^. It was found that cell clumps were also generated in Mp*GCAM1*-overexpressing Mp*rrb* knockout lines in this study (Fig. 5b). This indicates that cellular reprogramming requires MpGCAM1, but not the other downstream regulators of MpRRB. Nevertheless, neither the Mp*rra* knockout nor the overexpression of Mp*RRB* resulted in the formation of cell clumps^15^. This inconsistency was explained by a mild activation of cytokinin signaling while manipulating Mp*RRA* or Mp*RRB* expression. The Mp*rra* knockout removes the inhibitory effect of MpRRA but does not fully activate the MpRRB-mediated pathway, and the cytokinin signal upregulated by the overexpression of Mp*RRB* is alleviated by the negative feedback loop generated by MpRRB and MpRRA^15^. Indeed, our Mp*GCAM1*-overexpressing lines had Mp*GCAM1* transcripts at 10.7- to 19.3-fold of the wild-type (Fig. 5a), whereas Mp*rra* knockout and Mp*RRB*-overexpressing lines expressed Mp*GCAM1* at less than threefold (Fig. 2d,e).

It is noteworthy that, although morphologically indistinguishable, thalli that had regenerated from undifferentiated cell clumps were smaller in Mp*rrb* knockout lines compared to the wild-type (Fig. 5b). This is consistent with our previous observation that thallus growth was significantly inhibited in Mp*CKX2*-overexpressing lines^15^. Thalli are developed first through *de-novo* apical notch generation, and then by cell proliferation at the established notch. Although it remains unknown which process is affected by cytokinin signaling, the MpRRB-mediated pathway is probably involved in cell proliferation since cell differentiation undergoes properly during the development of Mp*rrb* thalli^15^. A few cell cycle regulators have been identified as the targets of the cytokinin signaling in *Arabidopsis*. For instance, the expression of cyclin D is under the control of B-type RRs^39^, and cytokinins promote the nuclear transport of 3R-type Myb transcription factors that induce a set of G2/M-specific genes^40^. These cell cycle regulators are also encoded in the *M. polymorpha* genome^12^. Thus, further studies are required to reveal the process of how cytokinins control them in non-vascular plants and whether the signaling cascade is conserved during evolution.

In the absence of functional *RAX* genes, axillary meristem formation is impaired in *Arabidopsis*; however, this defect can be recovered by exogenous cytokinin application or ectopic expression of *IPT8*^30^. The absence of Blind, a RAX ortholog in tomato, also causes a defect in axillary meristem formation due to insufficient cytokinin content^20,41^. RAXs are known to upregulate the expression of *SHOOT MERISTEMLESS* (*STM*), which then induces cytokinin biosynthesis genes and activates signaling, thereby elevating the expression of the *WUSCHEL* (*WUS*) homeobox gene^42^. On the other hand, the present study showed that, in *M. polymorpha*, MpGCAM1 does not regulate gemma cup formation through enhancing cytokinin biosynthesis (Fig. 6). Nevertheless, we occasionally observed gemma cup formation near the notch when one of the Mp*rra* Mp*gcam1* double knockout lines was treated with tZ (Fig. S4, line #15). We speculated that hyper-activation of cytokinin signaling by the tZ application in the absence of Mp*RRA* might have promoted gemma cup formation via unknown pathways, while it remains elusive whether such pathways indeed function under physiological conditions. Considering that, in *Arabidopsis*, the defects observed in the *rax1 rax2 rax3* triple mutant were partly rescued by the expression of Mp*GCAM1*^18^, it is likely that *RAX* orthologs have conserved their function in meristem formation by changing the promoter sequences of target genes during evolution.

Cytokinin-rich conditions alter the epigenetic status of the *Arabidopsis* genome during de novo shoot regeneration, and Type-B ARR can activate *WUS* transcription^43^. Therefore, it is intriguing how the epigenetic status is affected by cytokinins in *M. polymorpha*, and whether it controls organ growth and development. Future works using live-cell imaging and single-cell sequencing techniques will reveal how cytokinins coordinate cell division and differentiation at the apical notch and how the cytokinin signaling cascade has adapted in land plants to assist in coping with internal and external factors in distinct territories.

## Materials and methods

### Plant materials and growth conditions

The male and female *M. polymorpha* accessions, Takaragaike (Tak)-1 and Tak-2, respectively, were cultured on a half-strength Gamborg’s B5 agar medium under continuous white light at 22 °C. Mp*rra* and Mp*rrb* knockout lines, Mp*CKX2*-, Mp*RRA*- and Mp*RRB*-overexpressing lines^15^, and Mp*GCAM1pro:GUS* and Mp*gcam1* knockout lines^18^ were previously described. The Mp*gcam1* Mp*rra* double mutant was obtained by crossing the Mp*gcam1* line with the Mp*rra* line. Sexual organ formation was induced by culturing mature thalli under white light supplemented with far-red light^10^. All plant experiments involving *M. polymorpha* were carried out in accordance to relevant institutional, national, and international guidelines and legislation.

### GUS staining

pMpGWB103^44^ harboring the Mp*CKX2* cDNA was introduced into the Mp*GCAM1pro:GUS* line^18^ according to the method described by Kubota et al.^45^. GUS staining was performed as described by Althoff et al.^46^.

### Inducible overexpression of Mp*GCAM1*

Mp*GCAM1* cDNA were amplified by RT-PCR using total RNA prepared from Tak-1 thalli and the primers listed in Supplementary Table S3. This was followed by cloning in the plasmid pDONR221 using BP Clonase (Thermo Fisher Scientific, USA) to obtain a Gateway entry clone. Next, the Mp*GCAM1* cDNA was transferred to the destination vector pMpGWB313^44^ using LR Clonase (Thermo Fisher Scientific) in order to be fused with the Mp*EF1α* promoter and gene encoding glucocorticoid receptor (GR). The resulting plasmid was used for the transformation of Tak-2 or the Mp*rrb* knockout line. Thallus tips were cultured on an agar medium containing 5 µM of dexamethasone (DEX; FUJIFILM Wako Pure Chemical Corporation, Japan) to induce the expression of Mp*GCAM1*.

### qRT-PCR

To quantify the Mp*GCAM1* transcript level, thallus tips were incubated in a half-strength Gamborg’s B5 liquid medium for 24 h with shaking at 130 rpm. Next, trans-zeatin (Nacalai Tesque Inc., Japan) and/or cycloheximide (CHX; FUJIFILM Wako Pure Chemical Corporation) were added to the medium for a final concentration of 50 µM or 10 µM, respectively. This was followed by 2 or 3 more hours of incubation. To quantify the Mp*IPT1* and Mp*IPT2* transcript levels, 7-day-old thalli were incubated in a half-strength Gamborg’s B5 liquid medium for 24 h with shaking at 130 rpm. Then, DEX (FUJIFILM Wako Pure Chemical Corporation) was added to the medium at a final concentration of 10 µM, followed by 2-h incubation. For qRT-PCR analysis, total RNA was extracted with a FavorPrep Plant Total RNA Purification Mini Kit (Favorgen Biotech, Taiwan), and first-strand cDNAs were prepared using ReverTra Ace qPCR RT Master Mix with gDNA Remover (TOYOBO, Japan), according to the manufacturer’s instruction. qRT-PCR was performed with a THUNDERBIRD SYBR qPCR Mix (TOYOBO) and the LightCycler 480 Real-Time PCR System (Roche, Switzerland). The primers used for RT-PCR are listed in Supplementary Table S3.

### RNA-seq analysis

Using a RNeasy Plant Mini Kit, the total RNA was isolated from the tip region of mature thalli according to the manufacturer’s instruction (Qiagen, Netherlands) and was treated with DNase (Qiagen) to eliminate genomic DNA. Preparation of libraries and sequencing were performed by Macrogen Japan (Kyoto, Japan, http://www.macrogen-japan.co.jp/). Sequence reads were mapped to the Marchantia genome sequence v.3.1 by HISAT2 2.1.0^47^ and used to calculate FPKM values with Cuffdiff v.2.1.1^48^. A GO analysis was conducted with the Plant Transcriptional Regulatory Map (http://plantregmap.gao-lab.org/go.php)^49^.

## Supplementary Information

Supplementary Figures.

Supplementary Tables.

## Data Availability

The datasets generated and/or analyzed during the current study are available in the DDBJ Sequenced Read Archive under the Accession Numbers DRA014460.

## References

[1] Kieber JJ, Schaller GE (2014). Cytokinins. Arabidopsis Book.

[2] Hwang I, Sheen J (2001). Two-component circuitry in Arabidopsis cytokinin signal transduction. Nature.

[3] Sakai H (2001). ARR1, a transcription factor for genes immediately responsive to cytokinins. Science.

[4] Taniguchi M, Sasaki N, Tsuge T, Aoyama T, Oka A (2007). ARR1 directly activates cytokinin response genes that encode proteins with diverse regulatory functions. Plant Cell Physiol..

[5] To JP (2007). Cytokinin regulates type-A Arabidopsis response regulator activity and protein stability via two-component phosphorelay. Plant Cell.

[6] To JP (2004). Type-A Arabidopsis response regulators are partially redundant negative regulators of cytokinin signaling. Plant Cell.

[7] Mason MG (2005). Multiple type-B response regulators mediate cytokinin signal transduction in *Arabidopsis*. Plant Cell.

[8] Argyros RD (2008). Type B response regulators of *Arabidopsis* play key roles in cytokinin signaling and plant development. Plant Cell.

[9] Ishida K, Yamashino T, Yokoyama A, Mizuno T (2008). Three type- B response regulators, ARR1, ARR10 and ARR12, play essential but redundant roles in cytokinin signal transduction throughout the life cycle of *Arabidopsis thaliana*. Plant Cell Physiol..

[10] Ishizaki K, Nishihama R, Yamato KT, Kohchi T (2016). Molecular genetic tools and techniques for *Marchantia polymorpha* research. Plant Cell Physiol..

[11] Shimamura M (2016). *Marchantia polymorpha*: Taxonomy, phylogeny and morphology of a model system. Plant Cell Physiol..

[12] Bowman JL (2017). Insights into land plant evolution garnered from the *Marchantia polymorpha* genome. Cell.

[13] Suzuki H, Harrison CJ, Shimamura M, Kohchi T, Nishihama R (2020). Positional cues regulate dorsal organ formation in the liverwort *Marchantia polymorpha*. J. Plant Res..

[14] Flores-Sandoval E, Dierschke T, Fisher TJ, Bowman JL (2016). Efficient and inducible use of artificial microRNAs in *Marchantia polymorpha*. Plant Cell Physiol..

[15] Aki SS (2019). Cytokinin signaling is essential for organ formation in *Marchantia polymorpha*. Plant Cell Physiol..

[16] Aki SS, Nishihama R, Kohchi T, Umeda M (2019). Cytokinin signaling coordinates development of diverse organs in *Marchantia polymorpha*. Plant Signal. Behav..

[17] Schmülling T (2003). Structure and function of cytokinin oxidase/dehydrogenase genes of maize, rice, *Arabidopsis* and other species. J. Plant Res..

[18] Yasui Y (2019). GEMMA CUP-ASSOCIATED MYB1, an ortholog of axillary meristem regulators, is essential in vegetative reproduction in *Marchantia polymorpha*. Curr. Biol..

[19] Kato H, Yasui Y, Ishizaki K (2020). Gemma cup and gemma development in *Marchantia polymorpha*. New Phytol..

[20] Schmitz G (2002). The tomato blind gene encodes a MYB transcription factor that controls the formation of lateral meristems. Proc. Natl. Acad. Sci. U.S.A..

[21] Keller T, Abbott J, Moritz T, Doerner P (2006). Arabidopsis regulator of axillary *MERISTEMS1* controls a leaf axil stem cell niche and modulates vegetative development. Plant Cell.

[22] Müller D, Schmitz G, Theres K (2006). Blind homologous R2R3 Myb genes control the pattern of lateral meristem initiation in Arabidopsis. Plant Cell.

[23] Krapp A (2015). Plant nitrogen assimilation and its regulation: A complex puzzle with missing pieces. Curr. Opin. Plant Biol..

[24] Krapp A (2014). Nitrate transport and signalling in Arabidopsis. J. Exp. Bot..

[25] Yuan L (2007). The organization of high-affinity ammonium uptake in Arabidopsis roots depends on the spatial arrangement and biochemical properties of AMT1-type transporters. Plant Cell.

[26] Bhargava A (2013). Identification of cytokinin-responsive genes using microarray meta-analysis and RNA-Seq in Arabidopsis. Plant Physiol..

[27] Sakakibara H (2006). Cytokinins: Activity, biosynthesis, and translocation. Annu. Rev. Plant Biol..

[28] Brenner WG, Romanov GA, Köllmer I, Bürkle L, Schmülling T (2005). Immediate-early and delayed cytokinin response genes of *Arabidopsis thaliana* identified by genome-wide expression profiling reveal novel cytokinin-sensitive processes and suggest cytokinin action through transcriptional cascades. Plant J..

[29] Woodward AW, Bartel B (2005). Auxin: Regulation, action, and interaction. Ann. Bot..

[30] Wang J (2017). Cytokinin signaling activates *WUSCHEL* expression during axillary meristem initiation. Plant Cell.

[31] Wang Y (2014). The stem cell niche in leaf axils is established by auxin and cytokinin in *Arabidopsis*. Plant Cell.

[32] Kiba T, Kudo T, Kojima M, Sakakibara H (2011). Hormonal control of nitrogen acquisition: rolEs of auxin, abscisic acid, and cytokinin. J. Exp. Bot..

[33] Miyawaki K, Matsumoto-Kitano M, Kakimoto T (2004). Expression of cytokinin biosynthetic isopentenyltransferase genes in *Arabidopsis*: Tissue specificity and regulation by auxin, cytokinin, and nitrate. Plant J..

[34] Takei K (2004). *AtIPT3* is a key determinant of nitrate-dependent cytokinin biosynthesis in *Arabidopsis*. Plant Cell Physiol..

[35] Flores-Sandoval E, Eklund DM, Bowman JL (2015). A simple auxin transcriptional response system regulates multiple morphogenetic processes in the liverwort *Marchantia polymorpha*. PLoS Genet..

[36] Kato H (2015). Auxin-mediated transcriptional system with a minimal set of components is critical for morphogenesis through the life cycle in *Marchantia polymorpha*. PLoS Genet..

[37] Kato H (2020). Design principles of a minimal auxin response system. Nat. Plants.

[38] Mizuno Y (2021). Major components of the KARRIKIN INSENSITIVE2-dependent signaling pathway are conserved in the liverwort *Marchantia polymorpha*. Plant Cell.

[39] Riou-Khamlichi C, Huntley R, Jacqmard A, Murray JA (1999). Cytokinin activation of Arabidopsis cell division through a D-type cyclin. Science.

[40] Yang W (2021). Molecular mechanism of cytokinin-activated cell division in *Arabidopsis*. Science.

[41] Mapelli S, Lombardi L (1982). A comparative auxin and cytokinin study in normal and to-2 mutant tomato plants. Plant Cell Physiol..

[42] Wang Y (2021). Stem cell basis for fractal patterns: Axillary meristem initiation. Front. Plant Sci..

[43] Zhang TQ (2017). A two-step model for de novo activation of WUSCHEL during plant shoot regeneration. Plant Cell.

[44] Ishizaki K (2015). Development of Gateway binary vector series with four different selection markers for the liverwort *Marchantia polymorpha*. PLoS ONE.

[45] Kubota A, Ishizaki K, Hosaka M, Kohchi T (2013). Efficient *Agrobacterium*-mediated transformation of the liverwort *Marchantia polymorpha* using regenerating thalli. Biosci. Biotechnol. Biochem..

[46] Althoff F (2014). Comparison of the MpEF1α and CaMV35 promoters for application in *Marchantia polymorpha* overexpression studies. Transgenic Res..

[47] Pertea M, Kim D, Pertea GM, Leek JT, Salzberg SL (2016). Transcript-level expression analysis of RNA-seq experiments with HISAT, StringTie and Ballgown. Nat. Protoc..

[48] Trapnell C (2010). Transcript assembly and quantification by RNA-Seq reveals unannotated transcripts and isoform switching during cell differentiation. Nat. Biotechnol..

[49] Tian F, Yang DC, Meng YQ, Jin J, Gao G (2020). PlantRegMap: Charting functional regulatory maps in plants. Nucleic Acids Res..

