# Supplementary material for: R2R3-MYB transcription factor GEMMA CUP-ASSOCIATED MYB1 mediates the cytokinin signal to achieve proper organ development in Marchantia polymorpha
Source: Sci Rep. 2022 Dec 7;12:21123. doi: 10.1038/s41598-022-25684-3 (PMC9729187; doi:10.1038/s41598-022-25684-3)
Supplement: Supplementary file 1 — Supplementary Figures. [file 41598_2022_25684_MOESM1_ESM.pdf]

Supplementary Figure S1

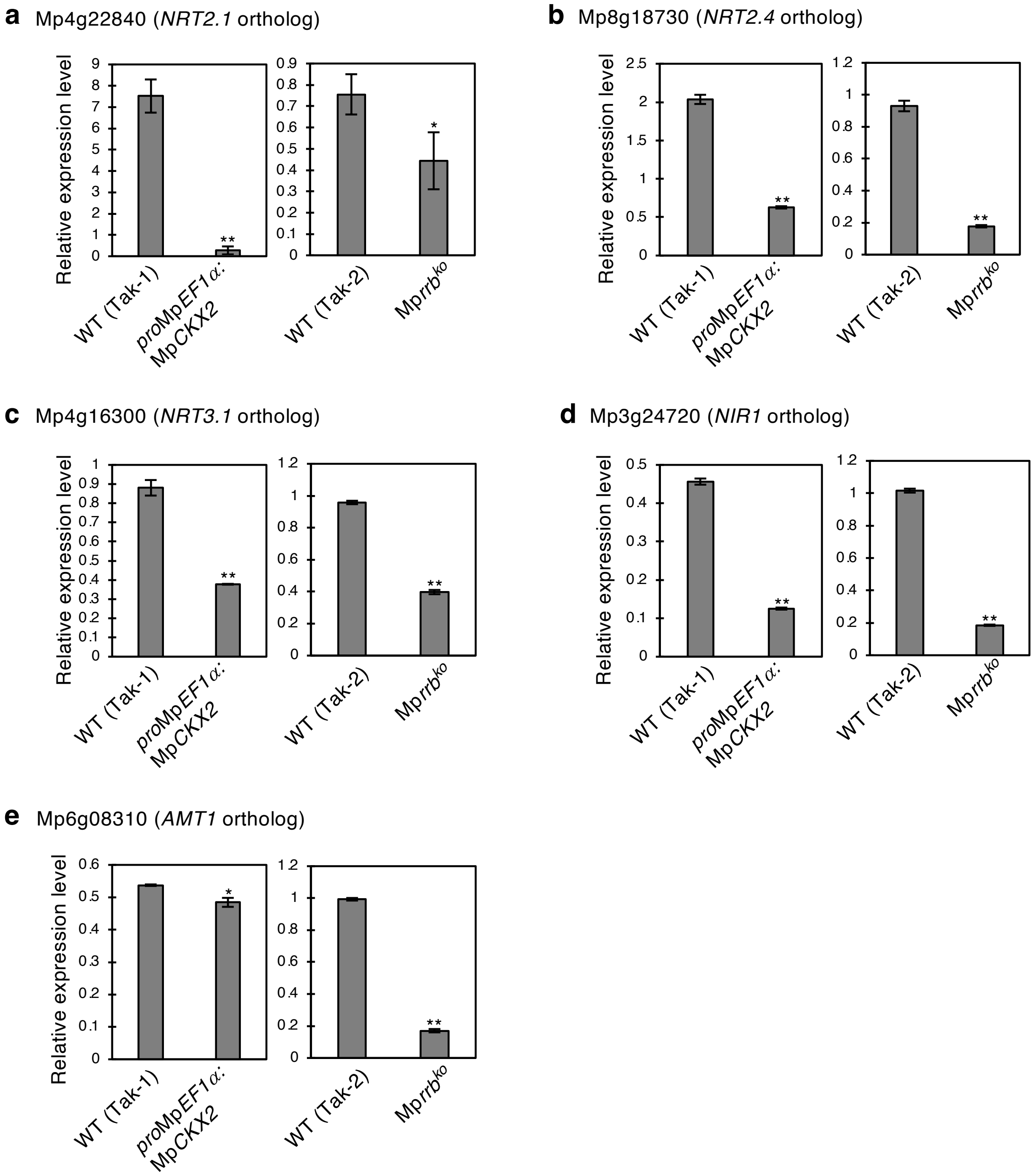

Supplementary Figure S1 Transcript levels of genes related to nitrate assimilation and transport in the Mp*CKX2*-overexpressing line and the Mp*rrb* knockout mutant. (a) Mp4g22840 (*NRT2.1* ortholog), (b) Mp8g18730 (*NRT2.4* ortholog), (c) Mp4g16300 (*NRT3.1* ortholog), (d) Mp3g24720 (*NIR1* ortholog), and (e) Mp6g08310 (*AMT1* ortholog) were examined by qRT-PCR. mRNA levels were normalized to that of Mp*EF1α*. Data are presented as mean ± SD ($n = 3$). Significant differences from the wild-type (Tak-1 or Tak-2) were determined by the Student's t-test as follows: *$P < 0.05$; **$P < 0.01$.

Supplementary Figure S2

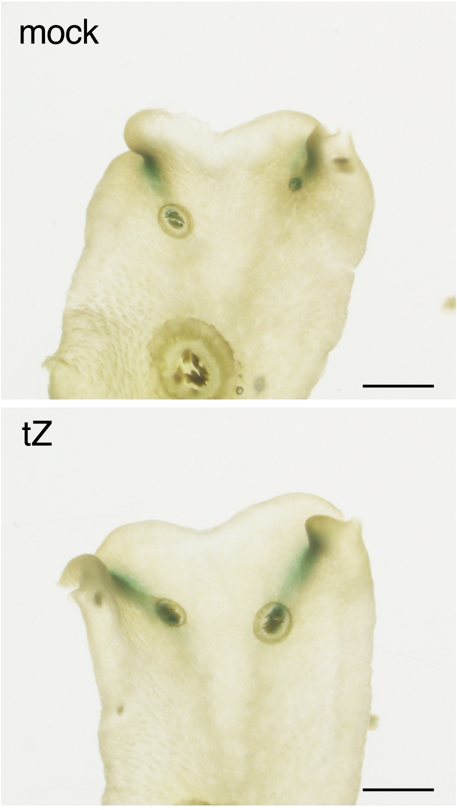

Supplementary Figure S2 Promoter activity of Mp*GCAM1* in the presence of exogenous cytokinin. 20-day-old thalli of Mp*GCAM1pro:GUS* were treated with or without 50 μM of tZ for four hours and subjected to GUS staining. Bar represents 2 mm.

Supplementary Figure S3

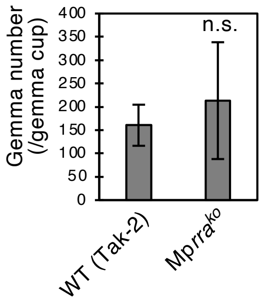

Supplementary Figure S3 Gemma number per gemma cup in the Mp*rra* knockout line. Data are represented as mean ± SD (n = 7). Significant difference from the wild-type (Tak-2) was determined by the Student's t-test: n.s., not significant.

Supplementary Figure S4

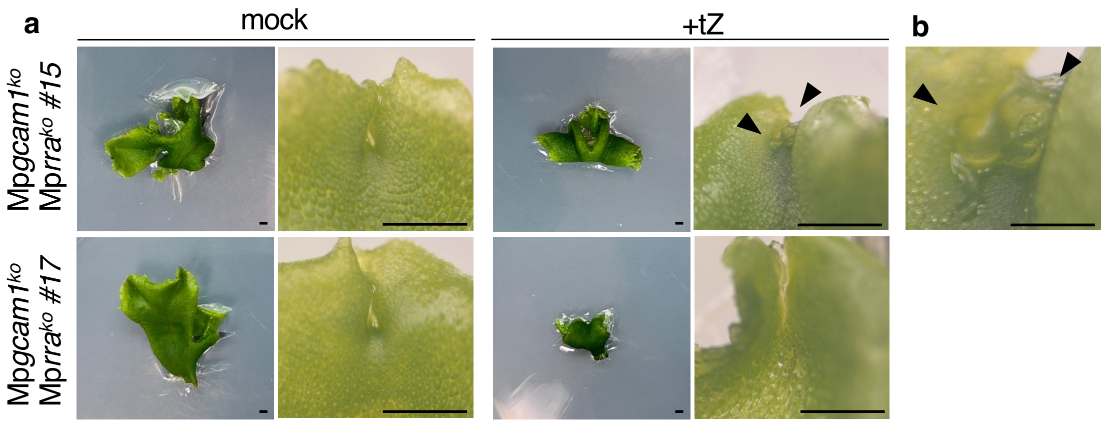

Supplementary Figure S4 Morphological phenotype of the Mp*gcam1* Mp*rra* double mutants grown in the presence of exogenous cytokinin. (a) Thallus apices of the two Mp*gcam1* Mp*rra* double mutants (#15 and #17) were treated with (+tZ) or without (mock) 50 µM tZ for 14 days. The right panels are magnified images of apical notches. Bars represent 2 mm. (b) Magnified image of gemma cups produced near the apical notch of tZ-treated plants (#15). Arrowheads indicate gemma cups. Bar represents 500 µm.
